# Reduced Cardiac Fructose 2,6 Bisphosphate Increases Hypertrophy and Decreases Glycolysis following Aortic Constriction

**DOI:** 10.1371/journal.pone.0053951

**Published:** 2013-01-07

**Authors:** Jianxun Wang, Jianxiang Xu, Qianwen Wang, Robert E. Brainard, Lewis J. Watson, Steven P. Jones, Paul N. Epstein

**Affiliations:** 1 Department of Pharmacology and Toxicology, University of Louisville, Louisville, Kentucky, United States of America; 2 Department of Physiology, University of Louisville, Louisville, Kentucky, United States of America; 3 Institute of Molecular Cardiology, University of Louisville, Louisville, Kentucky, United States of America; 4 Department of Pediatrics, University of Louisville, Louisville, Kentucky, United States of America; University of Southampton, United Kingdom

## Abstract

This study was designed to test whether reduced levels of cardiac fructose-2,6-bisphosphate (F-2,6-P_2_) exacerbates cardiac damage in response to pressure overload. F-2,6-P_2_ is a positive regulator of the glycolytic enzyme phosphofructokinase. Normal and Mb transgenic mice were subject to transverse aortic constriction (TAC) or sham surgery. Mb transgenic mice have reduced F-2,6-P_2_ levels, due to cardiac expression of a transgene for a mutant, kinase deficient form of the enzyme 6-phosphofructo-2-kinase/fructose-2,6-bisphosphatase (PFK-2) which controls the level of F-2,6-P_2_. Thirteen weeks following TAC surgery, glycolysis was elevated in FVB, but not in Mb, hearts. Mb hearts were markedly more sensitive to TAC induced damage. Echocardiography revealed lower fractional shortening in Mb-TAC mice as well as larger left ventricular end diastolic and end systolic diameters. Cardiac hypertrophy and pulmonary congestion were more severe in Mb-TAC mice as indicated by the ratios of heart and lung weight to tibia length. Expression of α-MHC RNA was reduced more in Mb-TAC hearts than in FVB-TAC hearts. TAC produced a much greater increase in fibrosis of Mb hearts and this was accompanied by 5-fold more collagen 1 RNA expression in Mb-TAC versus FVB-TAC hearts. Mb-TAC hearts had the lowest phosphocreatine to ATP ratio and the most oxidative stress as indicated by higher cardiac content of 4-hydroxynonenal protein adducts. These results indicate that the heart’s capacity to increase F-2,6-P_2_ during pressure overload elevates glycolysis which is beneficial for reducing pressure overload induced cardiac hypertrophy, dysfunction and fibrosis.

## Introduction

In the normal adult heart glucose accounts for only a small portion of cardiac energy supply, but in the failing heart glucose consumption increases and accounts for a greater fraction of cardiac fuel supply. This has been appreciated for over 40 years since Bishop and Altshuld reported [1] that glycolytic metabolism is increased in cardiac hypertrophy and congestive heart failure. However, the mechanism of increased glycolysis is uncertain. Nor is it certain if the elevation of cardiac glucose metabolism is an adaptive response of the failing heart or just another example of the reversion to fetal gene expression that occurs in hypertrophy and heart failure [2]. If it is an adaptive response to failure, then reducing the capacity of the heart to elevate glucose use should aggravate and potentially accelerate deterioration of the failing heart. Diabetes reduces glucose use and is a major risk factor for cardiac failure [3], suggesting that lower glucose metabolism plays a role in failure. However diabetes is a complex pathology and other changes to the diabetic heart may sensitize it to failure. Transgenic manipulations that increase [4] or decrease [5] cardiac glucose uptake have been used as a more targeted approach to testing the role of glucose usage in heart failure. However altered glucose uptake not only changes the contribution of glucose to cardiac energy supply, it may also alter activity of other glucose dependent pathways such as the pentose phosphate pathway, the hexosamine pathway, the polyol pathway and glycogen synthesis, which have all been implicated in cell injury [6]. An additional concern about relying on changing glucose uptake as a means to understand the role of elevated glycolysis in cardiac failure is that PET assays, performed on heart failure patients have not consistently reported increased glucose uptake in cardiac failure, thus suggesting that glucose uptake may not be key to up-regulating glycolysis in heart failure [7]. Therefore to understand the role and mechanism of increased cardiac glycolysis in heart failure it is necessary to alter glucose metabolism at other important metabolic steps that have been shown to be modified in heart failure.

Control of glycolysis is shared by several reactions [8]. One key reaction is carried out by 6-phosphofructo-1-kinase (PFK1) [9], [10]. Unlike glucose transport where there are two cardiac transporters that can compensate for one another, there is only a single PFK1 enzyme. PFK1 activity is tightly controlled. The most important positive regulator of PFK1 is fructose-2,6-bisphosphate (F-2,6-P_2_), which is increased in cardiac hypertrophy [11]. We previously described [12] a transgenic mouse called Mb which has reduced cardiac levels of F-2,6-P_2_ due to cardiac expression of a kinase deficient form of the enzyme 6-phosphofructo-2-kinase/fructose-2,6-bisphosphatase (PFK-2). The reduction of F-2,6-P_2_ in Mb transgenic hearts produces a reduction in glycolytic rate [12] due to reduced PFK1 activity. Because PFK1 is downstream of the glucose accessory pathways for pentose phosphate, hexosamine and glycogen synthesis it will have different effects on these pathways compared to reducing glucose uptake. These studies based on PFK1 inhibition will clarify our understanding of the importance of increased glycolysis in cardiac hypertrophy and heart failure.

Mb transgenic mice, which have reduced cardiac levels of F-2,6-P_2_ were subject to pressure overload by transverse aortic constriction (TAC) for 13 weeks to induce left ventricular hypertrophy. Reduced levels of F-2,6-P_2_ prevented the usual TAC induced rise in cardiac glycolysis and exacerbated cardiac dysfunction, hypertrophy and oxidative stress. These results indicate that the ability to up regulate F-2,6-P_2_ is an important adaptive response in the failing heart.

## Materials and Methods

### Ethics Statement

All animal procedures conformed to the National Institutes of Health *Guide for the Care and Use of Laboratory Animals* and were approved by the United State Department of Agriculture-certified institutional animal care committee of the University of Louisville (approval #11018). Surgeries were performed under avertin or ketamine/pentobarbital anesthesia. All efforts were made to minimize pain and analgesia was achieved with buprenorphine or ketoprofen.

### Mice

Mb transgenic mice were previously described [12] and express in cardiomyocytes the mutant enzyme kinase deficient, phosphatase active PFK-2 developed by Wu et al [13]. Mb and control mice were maintained on the background FVB. Male mice between ages 90–120 days were used for experiments. Animals were euthanized by cervical dislocation under anesthesia with avertin (0.4 g/kg).

### Transverse Aortic Constriction (TAC) Surgery

The TAC surgery was conducted by constriction of the transverse aorta as described [14]. Mice were anesthetized with avertin or ketamine (50 mg/kg, intra-peritoneal) and pentobarbital (50 mg/kg, intra-peritoneal), orally intubated with polyethylene-60 tubing, and ventilated with oxygen supplementation. An incision at the left second intercostal space was made to open the chest. A nylon suture was looped around the aorta between the brachiocephalic and left common carotid arteries. The suture was tied around a 27- gauge needle (put adjacent to the aorta) to constrict the aorta to a reproducible diameter. Then the needle was removed, leaving a discrete region of stenosis (TAC mice), and the chest was closed. Mice were extubated upon recovery of spontaneous breathing and were allowed to recover in warm, clean cages supplemented with oxygen. Analgesia (ketoprofen, 5 mg/kg or buprenorphine, 0.1 mg/kg) was given before mice recovered from anesthesia (and 24 and 48 hours later). Sham age-matched mice were subjected to the same procedure except the suture was only passed underneath the aorta and not tied off.

### Cardiac Perfusion for Measurements of Glycolysis

Langendorff perfusions were carried out as we previously described [15], [16]. The heart was rapidly cannulated through the aorta and retrogradely perfused at 2 ml/min with Krebs-Henseleit buffer (KH) consisting of 120 mM NaCl, 20 mM NaHCO_3_, 4.6 mM KCl, 1.2 mM KH_2_PO_4_, 1.2 mM MgCl_2_, 1.25 mM CaCl_2_, 5 mM glucose. Throughout the perfusion KH buffer was continuously equilibrated with 95% O_2_/5% CO_2_ which maintained a pH of 7.4 and temperature was maintained at 37°C. The heart was paced throughout the procedure at 6 Hz (6 V, 3 ms). For studying the effect of insulin, baseline glycolysis was determined for the first 30 min followed by 50 min in the presence of 200 µU/ml insulin. Glycolysis was measured with 5-^3^H-glucose as we previously described [12]. Tritiated water produced from tritiated glucose during cardiac perfusion was measured by diffusion and scintillation counting. Effluent from each time point of the perfusion was assayed in duplicate. For each experiment, background counts were determined by performing the same equilibration on perfusion buffer that had not passed through the heart. Diffusion efficiency was measured in each experiment using tritiated water.

### Echocardiographic Assessment of Cardiac Function

Transthoracic echocardiography of the left ventricle was performed using a 15-MHz linear array transducer (15L8) interfaced with a Sequoia C512 system (Acuson) as previously described [17]. Mice were anesthetized with 2% isoflurane, maintained under anesthesia with 1.25% isoflurane, and examined. Ventricular parameters were measured in M-mode with a sweep speed 200 mm/s. The echocardiograms were captured from short-axis views of the left ventricle (LV) at the midpapillary level. LV percent fractional shortening (LV%FS) was calculated according to the following equation: LV%FS = [(LVEDD-LVESD)/LVEDD]×100. All data were calculated from 10 independent cardiac cycles per experiment.

### Quantitative Real Time Polymerase Chain Reaction (RT-PCR)

Cardiac RNA was extracted with Trizol reagent. The total RNA was transcribed to cDNA with Superscript II enzyme and random oligonucleotide primers (Invitrogen). The primers, probes and reaction buffer for RT-PCR were purchased from AB (Applied Biosystem) including hOGG1(Hs00213454_m1), α-MHC (Mm01313844_mH), β-MHC(Mm00600555_m1), ANP (Mm01255748_g1), BNP (Mm01255770_g1), procollagen 1α1(Mm01302043_g1), procollagen 3α1(Mm01254476_m1), 18S RNA (Hs99999901_s1) and 2× Master buffer. RT-PCR was carried out on AB 7300 thermocycler with 35 cycles, each cycle consisted of 95°C for 15 seconds, 55°C for 15 seconds and 75°C for 30 seconds. Ribosomal 18S RNA was used as endogenous control. Relative abundance of transcripts was determined by the delta-delta CT method. The choice of a control gene for normalizing RT-PCR results is important to obtain maximal precision for calculating target gene expression. Expression of individual housekeeping genes is not always constant under different experimental conditions. Ribosomal RNA standards such as 18S are far more abundant than any target mRNA which can influence background subtraction [18]. Thus the use of 18S RNA as the sole RT-PCR standard in the current study is likely to reduce precision and is less accurate than use of multiple housekeeping mRNAs as standard [18].

### Histological Experiments

Cryostat sections (5 µm) were fixed in 10% formalin for 15 min and washed three times with PBS. The cryostat slides were incubated with a saturated solution of picric acid containing 0.1% Sirius red for staining collagen and 0.1% fast green for staining noncollagen proteins. Staining was performed in the dark for 2 hours. The slides were then rinsed with distilled water, dehydrated with alcohol, and mounted with Permount. The sections were visualized and photographed by a blinded observer. Interstitial fibrosis in the sections was scored by a blinded observer against reference images using a scale of 1 to 4 based on the severity of fibrosis with scores of 1 for low, 2 for mild, 3 for moderate, and 4 for severe.

### Western Blots

Immunoblots were performed as previously described [19] with modification. In brief, frozen cardiac tissue was homogenized with lysis buffer containing 50 mmol/L Tris-HCl (pH7.5), 5 mmol/L EDTA, 10 mmol/L EGTA, 1× cock tail protease inhibitor, 1× alkaline phosphatase inhibitor and 1× acid phophatase inhibitor, 50 ug/ml phenylmenthysulfonyl fluoride and 1.23 mg/ml Chaps. Extracts were centrifuged at 12000 rpm at 4°C for 15 minutes. The protein concentration was determined by Lowry method (Pierce). 10 ug of the sample proteins was mixed with loading buffer (40 mmol/L Tris-HCl, pH 6.8, 1% SDS, 50 mmol/L DTT, 7.5% glycerol and 0.003% bromophenol blue and heated at 95°C for 5 minutes, and subjected to electrophoresis on a gradient gel (4% to 12%, Invitrogen) at 120V. After electrophoresis, the protein was transferred to a PVDF membrane in a transfer buffer (Invitrogen). The PVDF membrane was rinsed briefly in TBS buffer containing 50 mM Tris, 137 mM NaCl, pH 7.5 and blocked in buffer (5% milk with 0.5% BSA in TBST buffer (TBS buffer containing 0.1% tween 20) at room temperature for 1 hour. The membrane was then incubated with rabbit anti 4-hydroxy-2-noneal (4HNE) antibody at 1∶3000 dilution(Abcam ) at 4°C over night, followed by washing three times. The secondary antibody was incubated with the membrane for another one hour at room temperature. Finally the antigen-antibody complexes were visualized with use of an enhanced chemiluminescence (ECL, GE Healthcare) kit. Anti-GAPDH (Abcam) was used for normalizing.

### Metabolites Measurement

For the assay of ATP and phosphocreatine, freeze clamped hearts were powdered in liquid nitrogen and weighed. Powered samples were homogenized in 1 M ice-cold perchloric acid and centrifuged. Supernatants were neutralized with 2 M KHCO_3_.The supernatant from neutralized extracts was used for the estimation of metabolites by fluorometric procedures [20].

For assay of F-2,6-P_2_, freeze clamped heart tissue was homogenized with 10–20 volumes of 50 mM NaOH and kept at 80°C for 5 min. The extract was cooled on ice and neutralized by adding 1 M acetic acid with 20 mM HEPES. After centrifugation at 8000 g for 10 min, the supernatant was collected for F-2,6-P_2_ by the PFK1 activation method [21], [22].

Pyridine nucleotides were measured by spectrophotometric enzymatic cycling method for both the oxidized and reduced forms of NADP(H) and NAD(H) [23], [24] with modification. Briefly, freeze clamped hearts were homogenized with 30 volumes of cold 40 mM NaOH containing 0.5 mM cysteine buffer for 30 seconds. After centrifuging at 10,000 rpm for 15 min, the supernatant was divided into two parts. One part was heated at 60°C for 20 min to destroy all NAD(P) for assay of NADH or NADPH, the other part was kept on ice for assay of total NADP(H) or NAD(H). The difference between total NADP(H) and NADPH is NADP^+^and the difference between total NAD(H) and NADH is NAD^+^. For NAD(H) assay, the buffer containing 100 mM Tris-HCl, pH 8.0, 2 mM PES (phenazine ethosulfate), 0.5 mM MTT (3-(4,5-dimethylthiazolyl-2)-2,5-diphenyltetrazolium bromide), 0.2 mg/ml ADH (alcohol dehydrogenase ) and 600 mM alcohol. For assay of NADP(H), the buffer containing 100 mM Tris-HCl pH 8.0, 5 mM EDTA, 2 mM PES, 0.5 mM MTT, 1.3 U/ml G6PDH (glucose-6-phosphate dehydrogenase) and 1.0 mM G-6-P (glucose-6-phosphate). The reaction mixtures were kept at room temperature for 15 min, and then read by spectrophotometer at 560 nm.

### Statistical Analysis

Statistical comparisons were performed by two-way ANOVA using Sigma Stat software with sham or TAC surgery as one factor and transgenic or non-transgenic as the other factor. The accepted level of significance was 0.05.

## Results

### TAC Induced Changes in F-2,6-P_2_ and Glycolysis

**Figure 1:**
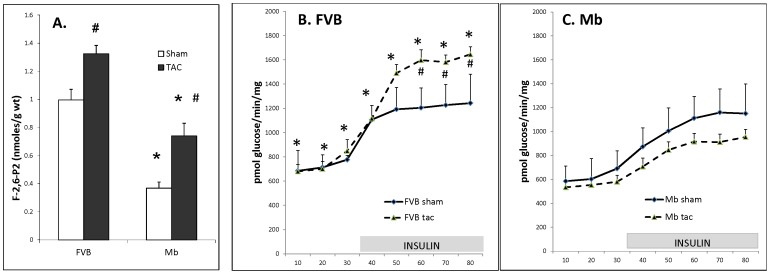
Cardiac F-2,6-P_2_ content and glycolysis in FVB and Mb hearts 13 weeks after sham or TAC surgery. Panel A shows that Mb F-2,6-P_2_ content was reduced relative to FVB under both sham and TAC conditions (* indicates P≤0.05). TAC treatment increased F-2,6-P_2_ content relative to sham in both groups (# indicates P≤0.05). Panels B and C show that in the presence of insulin TAC increased glycolysis in FVB hearts (# indicates P≤0.05) but did not in Mb hearts. After TAC treatment glycolytic rate was higher at all time points in FVB-TAC hearts (panel B) compared to Mb-TAC hearts (panel C) (* indicates P≤0.05 for FVB-TAC versus Mb-TAC ). Hearts were assayed for F-2,6-P_2_ content or glycolysis as described in the Methods Section. For F-2,6-P_2_ content 5 to 8 hearts were used per group. For glycolysis assays 6 FVB-sham, 6 Mb-sham, 9 FVB-TAC and 16 Mb-TAC hearts were measured per group. Data was analyzed by 2-way ANOVA using surgery and mouse type as factors. FVB and Mb glycolysis results are shown on 2 separate graphs for clarity. Axes are the same on both graphs.

Mb transgenic mice were originally created [12] to decrease glycolysis by reducing the level of F-2,6-P_2_. This study confirmed the reduction in F-2,6-P_2_ in hearts from Mb-sham mice (Figure 1A). Thirteen weeks after TAC surgery F-2,6-P_2_ content increased in FVB and Mb mice. Despite the increase in F-2,6-P_2_ levels of Mb-TAC hearts their F-2,6-P_2_ content was still lower than in FVB-sham or FVB-TAC hearts.

Glycolysis was measured in Langendorff perfused hearts from all 4 groups. In FVB hearts (Figure 1B) TAC treatment increased glycolysis in the presence of insulin and this difference was significant at the last 3 time points. In contrast to FVB results, TAC treatment did not increase glycolysis in Mb hearts (Figure 1C) and glycolysis was actually slightly lower at all 8 time points in Mb-TAC hearts relative to Mb-sham hearts. At all time points during the perfusion FVB-TAC glycolysis was significantly higher than Mb-TAC glycolysis (compare the dashed lines in Figures 1B and 1C, which have the same axes to enable comparisons between the 2 graphs).

### Cardiac Structural and Functional Changes after TAC Surgery

**Figure 2:**
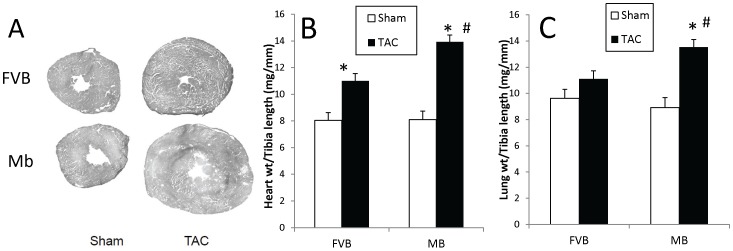
The Mb transgene exacerbates TAC induced increases in cardiac hypertrophy and lung weight. Mice were sacrificed 13 weeks after surgery. Panel A shows representative transverse cardiac cross-sections. The graphs in panels B and C show heart weight and lung weight to tibia length, respectively. Mb-TAC hearts and lungs weighed more than FVB-TAC organs. *p<0.01, sham vs. TAC; #p<0.05, Mb-TAC vs. FVB TAC by two way ANOVA, n = 20 for FVB-sham, n = 22 for FVB-TAC, n = 16 for Mb-sham and n = 26 for Mb-TAC.

The functional and structural response to TAC surgery were assessed to determine whether the lower F-2,6-P_2_ and glycolytic response of Mb hearts made them more sensitive to pressure overload than FVB hearts. Thirteen weeks after surgery, both groups of TAC hearts were significantly enlarged compared to sham hearts (Figure 2A and B). There was no significant difference between Mb-sham and FVB-sham hearts but Mb-TAC hearts were larger than FVB-TAC hearts (p<0.05). Heart weight to tibia length ratio increased by 37% in FVB-TAC mice and by 72% in Mb-TAC mice compared to sham mice of the same genotype. The results indicate that decreased glycolysis in Mb mice is associated with greater hypertrophy in response to pressure overload. An increase in the ratio of lung weight to tibia length is a feature of heart dysfunction due to pulmonary fluid accumulation. Thirteen weeks after TAC surgery FVB mice did not exhibit a significant increase in this parameter. In contrast, Mb mice showed an (P<0.05) elevation of lung weight to tibia length ratio (Figure 1D) which was 36% higher than the ratio in FVB-TAC mice. This reinforces the impression that the Mb transgene makes the heart more susceptible to pressure overload induced cardiac dysfunction.

Echocardiography was used to assess cardiac structural and functional changes (Table 1). Left ventricular end diastolic diameter (LVEDD) and left ventricular end systolic diameter (LVESD) were significantly increased in Mb-TAC and FVB-TAC compared to the corresponding sham hearts. Both LVEDD and LVESD were greater in Mb-TAC compared to FVB-TAC hearts. Fractional shortening (FS) was reduced by TAC and to a greater extent in Mb-TAC hearts than in FVB-TAC hearts. Thus, in addition to hypertrophy, the Mb transgene was associated with a more impaired functional response to TAC.

**Table 1:** Cardiac function measured by echocardiography 13 weeks after TAC or sham surgery.

Parameter	FVB sham	FVB TAC	Mb sham	Mb TAC
**BW (g)**	30.4±0.8	30.8±0.8	29.3±0.7	30.6±0.9
**LVEDD (mm)**	3.81±0.08	4.15±0.05^a^	3.97±0.08	4.40±0.08a,b
**LVESD (mm)**	2.36±0.10	2.76±0.08^a^	2.48±0.07	3.17±0.09a,b
**IVS (D) (mm)**	0.91±0.02	1.02±0.03^a^	0.86±0.03	1.03±0.02^a^
**IVS (S) (mm)**	1.18±0.03	1.29±0.03^a^	1.13±0.02	1.25±0.02^a^
**PWTh (D)(mm)**	0.80±0.04	0.89±0.03^a^	0.74±0.02	0.88±0.03^a^
**PWTh (S)(mm)**	1.16±0.04	1.25±0.02^a^	1.11±0.04	1.25±0.05^a^
**IVS%Th**	30.9±2.8	29.3±3.5	33.6±3.6	24.1±2.26
**PW%Th**	40.7±5.2	40.3±3.8	44.7±1.9	42.0±4.0
**IVS/PW**	1.16±0.05	1.13±0.03	1.15±0.04	1.15±0.05
**%FS**	38.6±1.7	33.8±1.1^a^	37.4±1.3	27.1±1.1a,b
**HR**	487±10	516±11	533±7	525±16

Legend: Data are mean±SE.

a indicates p<0.05 for FVB-sham vs. FVB-TAC and for Mb-sham vs. Mb-TAC;

b indicates p<0.05 for Mb-TAC vs. FVB-TAC, n = 8 for sham groups, n = 12 for TAC groups. LVEDD, left ventricular end diastolic diameter; LVESD, left ventricular end systolic diameter; IVS(D), interventricular septum thickness at diastole; IVS(S), interventricular septum thickness at systole; PWTh(D), post wall thickness at diastole; PWTh(S), post wall thickness at systole; IVS%Th, interventricular septum % thickening; PW%Th, posterior wall % thickening; IVS/PW, interventricular septum to posterior wall thickness ratio; %FS, percent fractional shortening; HR, heart rate. Data were analyzed by two way ANOVA.

**Figure 3:**
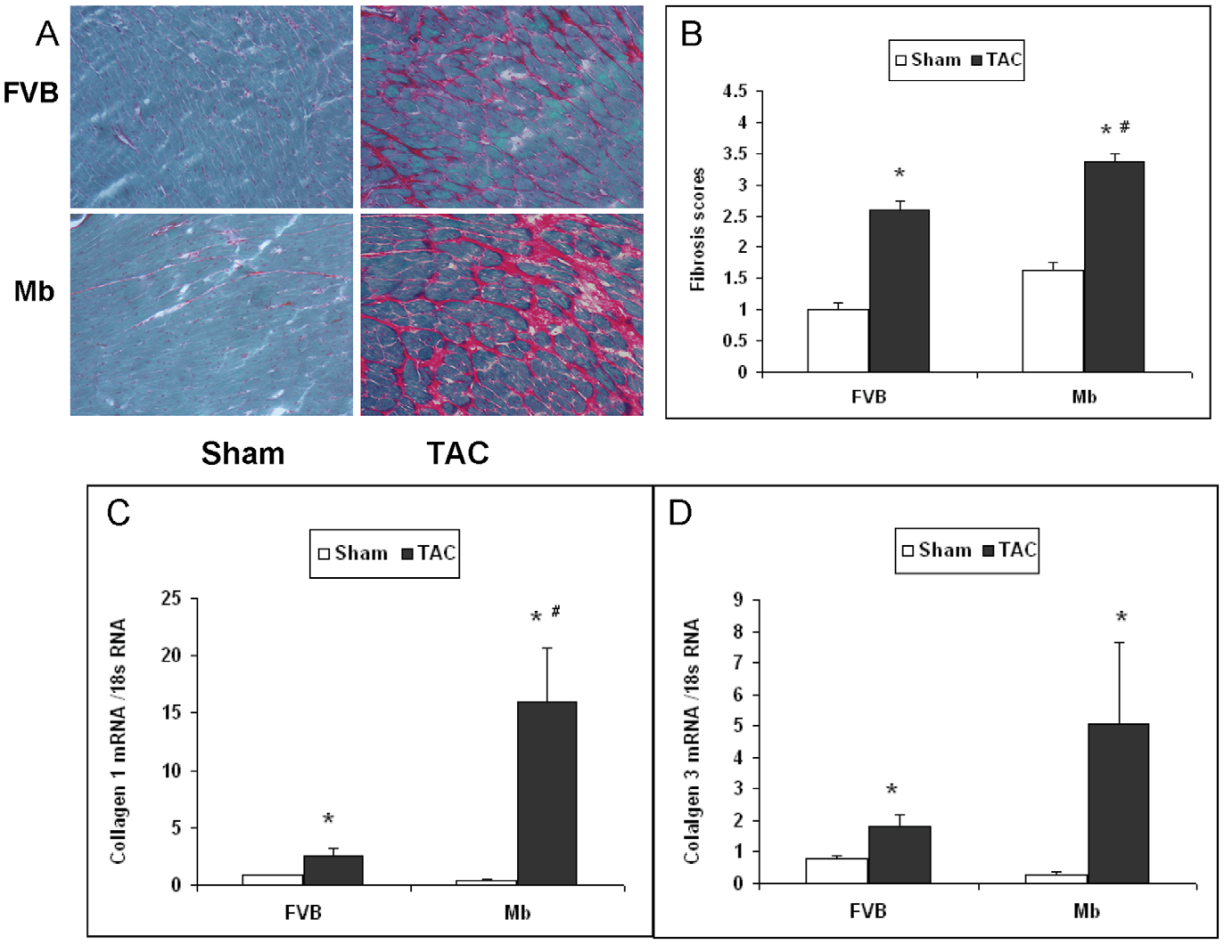
Fibrosis is increased in Mb hearts 13 weeks after TAC. (A) Representative sirius red staining for fibrosis in FVB and Mb hearts. (B) Semiquantitative scores for fibrosis staining performed as described in Methods by a blind observer. Staining of fibrosis in Mb-TAC hearts was significantly higher than that in FVB-TAC hearts. The expression of collagen 1 mRNA (panel C) measured by quantitative RT-PCR was significantly higher in Mb-TAC hearts than in FVB-TAC hearts. Expression of collagen 3 mRNA (panel D) was elevated in TAC hearts but not significantly different in Mb-TAC versus FVB-TAC hearts. #P<0.05, Mb-TAC vs. FVB-TAC and *P<0.05, Sham vs. TAC by 2-way ANOVA, n = 5 for each group.

Blind, semiquantative analysis of sirius red staining indicated that fibrosis was increased 13 weeks after TAC surgery (Figures 3 A and B, P<0.05) in both Mb and FVB mice. However the fibrosis staining was significantly greater in Mb-TAC mice compared to FVB-TAC mice (P<0.05). In part the greater fibrosis score in Mb-TAC mice may have been due to higher basal fibrosis in Mb-sham hearts compared to FVB-sham hearts and the TAC induced increase in fibrosis score was not significantly greater in Mb hearts than in FVB hearts. However, the conclusion that TAC increased fibrosis to a greater extent in Mb hearts was supported by quantitative RT-PCR assays of cardiac collagen 1 mRNA expression (Figure 3C). Compared to FVB-TAC mice, expression of collagen 1 mRNA was 5-fold higher in Mb-TAC mice. Collagen 3 mRNA was not significantly different in FVB-TAC and Mb-TAC mice. A potential concern for all of the RT-PCR studies in this paper is that the assays were performed using ribosomal 18S RNA to normalize target mRNA expression. As previously published [18] and noted in the Material and Methods section, 18S RNA is far more abundant than any mRNA species which may adversely influence calculations of precise RT-PCR data.

**Figure 4:**
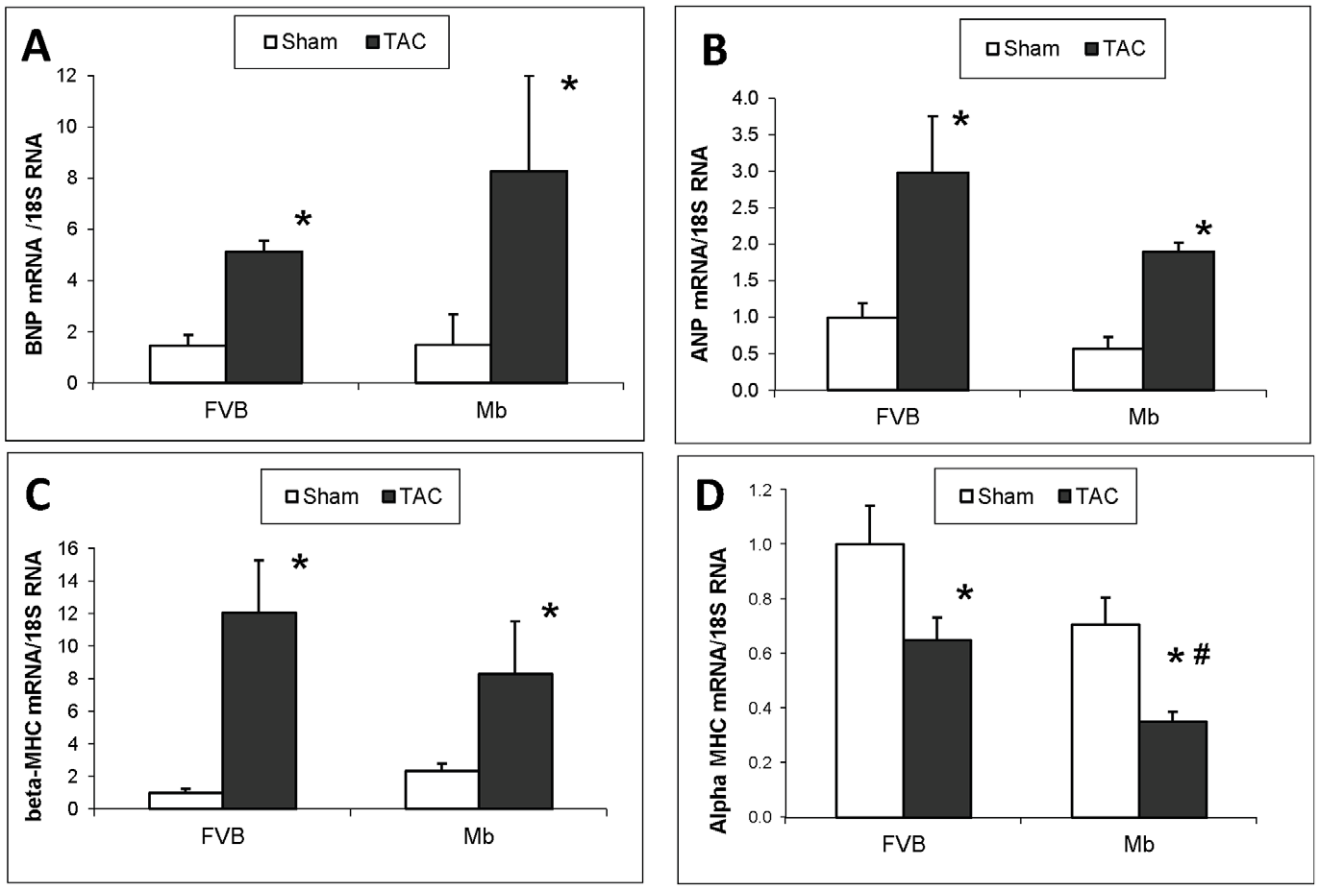
Hypertrophic biomarkers in FVB and Mb hearts 13 weeks after TAC or sham surgery. BNP (A), ANP (B), β-MHC (C) and α-MHC (D) mRNAs analyzed by quantitative RT-PCR. Expression of all markers was altered by TAC surgery. Only α-MHC mRNA was significantly different in Mb-TAC compared to FVB-TAC hearts. *p<0.05, sham vs. TAC; #p<0.05, Mb TAC vs. FVB TAC by two way ANOVA, n = 5 per group.

Hypertrophic biomarkers BNP, ANP, β-MHC and α-MHC mRNAs were compared by quantitative RT-PCR (Figure 4). Expression of each of these biomarkers was significantly changed by TAC surgery. The difference in BNP, ANP and β-MHC mRNA content in TAC treated Mb and FVB mice did not reach significance, whereas expression of α-MHC mRNA was significantly lower in Mb-TAC mice compared to FVB-TAC mice (P<0.05).

### TAC Induced Changes in Energy Reserves

**Figure 5:**
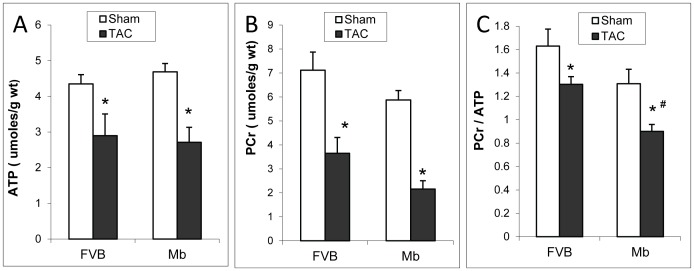
ATP and phosphocreatine in FVB and Mb hearts after TAC or sham surgery. Panels A–C show ATP, phosphocreatine (PCr) and the ratio of PCr to ATP. TAC decreased the content of ATP (A) and PCr (B), however, the differences between FVB and Mb were not significant. TAC decreased the ratio of PCr to ATP (C) in both FVB and Mb mice, moreover, a significant decrease of the ratio was found in Mb-TAC comparing to FVB-TAC. Statistical analysis was done by two way ANOVA, * indicates p<0.05, sham vs. TAC; # indicates p<0.05, Mb-TAC vs. FVB-TAC. n = 5 for FVB-sham, Mb-sham and FVB-TAC, n = 8 for Mb-TAC.

The ratio of phosphocreatine (PCr) to ATP correlates with the degree of heart failure [25] and is a prognostic indicator of survival [26]. ATP and PCr were reduced by TAC surgery in both types of mice (Figures 5A and B). The ratio of PCr to ATP was significantly lower in Mb-TAC hearts compared to FVB-TAC hearts (Figure 5C). This suggests more impaired cardiac energy reserves in Mb-TAC mice.

### The Changes of Redox Status Associated with the Changes of Content of Pyridine Nucleotides

**Figure 6:**
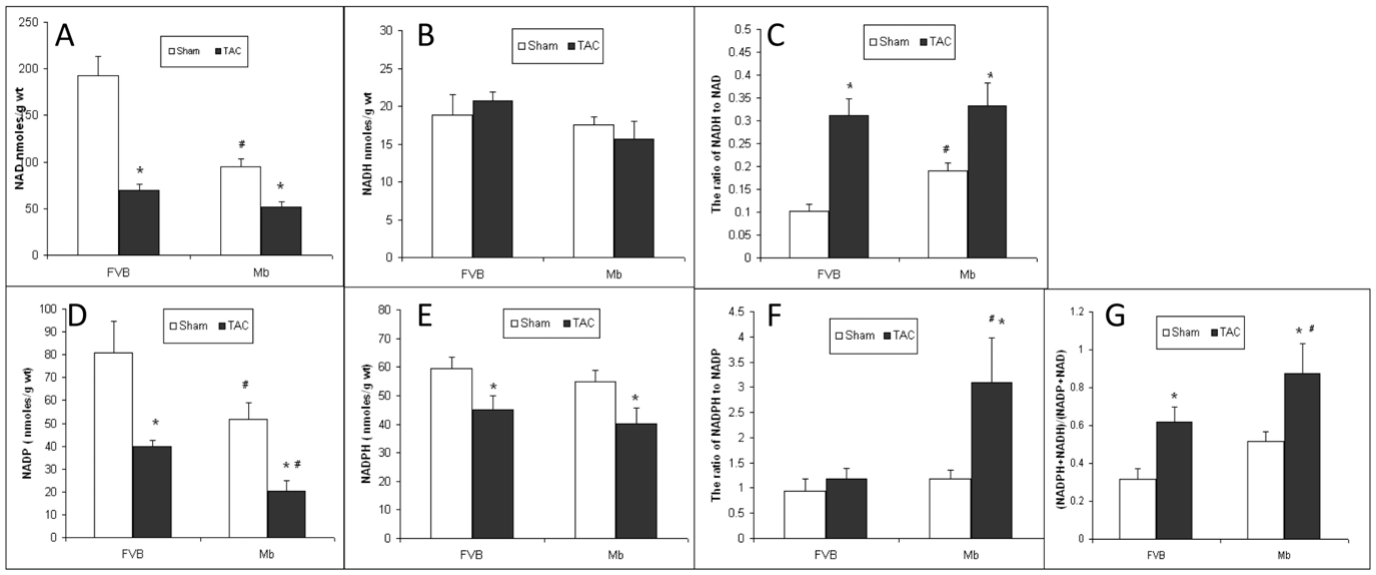
The content of pyridine nucleotides in FVB and Mb hearts. (A) The content of NAD in Mb-sham was significantly lower than in FVB-sham. After TAC, NAD content decreased significantly in both FVB and Mb hearts, whereas no significant difference was found between FVB-TAC and Mb-TAC. (B) NADH content was similar in FVB and Mb hearts in sham and TAC groups. (C) The ratio of NADH to NAD was significantly higher in Mb-sham than in FVB sham. After TAC, the ratio in FVB and Mb went up significantly, whereas no significant difference was found when comparing FVB-TAC with Mb-TAC. (D) NADP+ content was lower in Mb-sham compared to FVB-sham. TAC decreased NADP+ content in both FVB and Mb hearts and NADP+ content in Mb-TAC was significantly lower than in FVB-TAC. (E) A significant decrease of NADPH content was observed in FVB and Mb hearts after TAC. The NADPH content was constant in FVB-sham and Mb-sham while no significant difference was found between FVB-TAC and Mb-TAC. (F) TAC increased the ratio of NADPH to NADP+ in Mb but not in FVB hearts. Furthermore, the ratio in Mb-TAC was significantly higher than in FVB-TAC. (G) The ratio of reduced pyridine nucleotides, NADPH plus NADH to oxidized nucleotides, NADP+ plus NAD+ was significantly increased in both FVB and Mb hearts after TAC, and the ratio in Mb-TAC was significantly greater than in FVB-TAC. * indicates p<0.05 for sham vs. TAC; # indicates p<0.05 for Mb-sham vs. FVB-sham or Mb-TAC vs. FVB-TAC by two way ANOVA, n = 8 for Mb-TAC and n = 5 for other groups.

NADH is a reducing agent produced primarily by glycolysis in the cytosol and by the TCA cycle in mitochondria. NAD^+^ is the oxidized form of NADH. The ratio of NADH to NAD reflects the cellular redox status. NAD^+^ content was reduced by TAC surgery and was significantly higher in FVB-sham than in Mb-sham (Figure 6A). NADH levels were similar in all groups (Figure 6B). TAC increased the ratio of NADH/NAD^+^ in FVB and Mb mice and this ratio was similar in both TAC groups (Figure 6C). Due to lower NAD^+^ content in Mb-sham mice, the NADH/NAD^+^ ratio was higher in Mb-sham than in FVB-sham mice.

NADPH is a reducing agent and important for decreasing oxidative stress. NADP^+^ is the oxidized form of NADPH. NADP^+^ content was significantly reduced by TAC and was lower in Mb hearts than in FVB hearts for sham and TAC treatment (Figure 6D). TAC decreased NADPH content in Mb and FVB mice but there were no significant differences between Mb and FVB groups (Figure 6E). The ratio of NADPH to NADP^+^ in FVB-TAC did not show a statistical difference compared to FVB sham. In contrast, the ratio of NADPH to NADP^+^ in Mb-TAC increased 3 fold compared to Mb-sham and FVB-TAC (Figure 6F). This was primarily due to the very low NADP^+^ content in Mb TAC hearts.

An indicator of reductive status can be calculated as the ratio of total NADPH plus NADH to total NADP^+^ plus NAD^+^ (NAD(P)H/NAD(P)^+^), shown in Figure 6G. TAC increased this ratio in both FVB and Mb hearts and the ratio was significantly higher than in FVB TAC. Therefore, TAC increased reductive status and Mb hearts exhibited a more reductive status after TAC than FVB hearts.

### Oxidative Stress Levels in FVB and Mb after TAC

**Figure 7:**
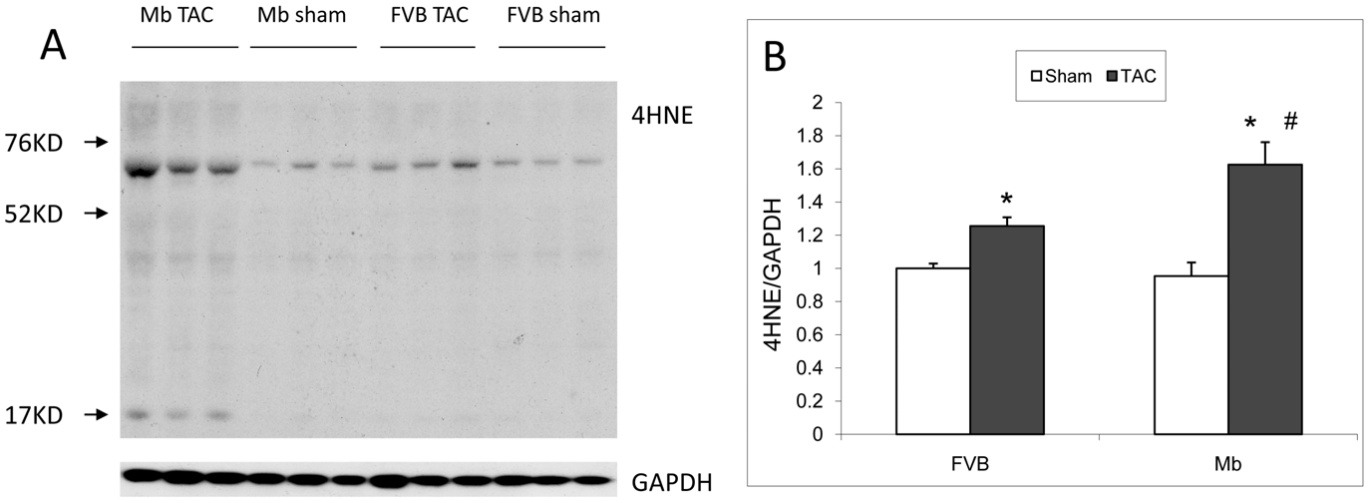
Oxidative stress shown by 4HNE adducts in FVB and Mb hearts. (A) Representative western blot showing 4HNE adducts and GAPDH loading control. (B) Statistical results of 4HNE adducts normalized by GAPDH. TAC increased the 4HNE adducts in both FVB and Mb hearts. Mb-TAC exhibited more 4HNE adducts than FVB-TAC. * indicates p<0.05 for sham vs. TAC; # indicates p<0.05 for Mb-TAC vs. FVB-TAC by two way ANOVA analysis, n = 6 for each group.

The protein adduct 4-hydroxynonenal (4HNE) is a marker of lipid peroxidation and oxidative stress. 4HNE western blots (Figure 7) were used to evaluate oxidative stress levels in hearts. TAC increased the level of 4HNE adducts in both FVB and Mb hearts. 4HNE was significantly higher in Mb-TAC than in FVB-TAC hearts.

## Discussion

PFK1 enzyme carries out one of the rate limiting steps of glycolysis. The metabolite F-2,6-P_2_ is a potent activator of PFK1 [21], [22]. We previously demonstrated that Mb transgenic hearts have reduced levels of F-2,6-P_2_ and decreased cardiac glycolysis [12]. TAC surgery increases cardiac glycolysis at least in part by raising F-2,6-P_2_ content [11]. In the current study, TAC increased F-2,6-P_2_ content in both FVB control and Mb transgenic hearts. However, the transgene for kinase deficient PFK2 in Mb hearts limited the rise in TAC induced F-2,6-P_2_. As a result cardiac glycolysis did not increase in TAC treated Mb mice and cardiac damage was greatly exacerbated. The results imply that stimulation of PFK2 and elevation of F-2,6-P_2_ are key adaptive responses to cardiac pressure overload.

The mechanism for PFK2 activation during cardiac stress involves the energy sensing enzyme AMP-activated protein kinase (AMPK) [27]. PFK2 is one of many AMPK substrates. AMPK phosphorylates and activates PFK2 kinase, producing a rise in F-2,6-P_2_. This increases the rate of glycolysis by stimulating PFK1. Heart failure increases expression of AMPK subunits in mouse and human cardiac samples [28]. Furthermore, if the AMPK response is inhibited by knockout of the AMPK alpha2 subunit, TAC induced pressure overload produces more severe cardiac dysfunction, hypertrophy and fibrosis [29] but the knockout has no effect on unstressed hearts. The AMPK knockout results are analogous to the current results obtained in Mb transgenic mice and suggest that F-2,6-P_2_ is a mediator of the protective effect of cardiac AMPK.

Cardiac specific reduction of glycolysis in Mb hearts resulted in more hypertrophy and more severe fibrosis after TAC. These findings are consistent with a study in another model of decreased cardiac glycolysis, the Glut4 knockout, which also exhibited cardiac hypertrophy and increased fibrosis [5]. Hypertrophic growth in the heart is mediated by growth factor pathways that increase protein synthesis, induce enlargement of cardiomyocytes and promote reorganization of sarcomeres within individual cardiomyocytes. The growth factor pathways are stimulated by mechanical stress on the heart. Decreased glycolysis in Mb mice or Glut4 knockout mice might sensitize the heart to growth factors and/or might sensitize the heart to synthesize and secrete more of these growth factors.

Mb-TAC hearts demonstrated more impaired cardiac function than FVB-TAC hearts. This was manifest as a decrease in FS, a higher ratio of lung weight to tibia length and lower production of ATP. Cardiomyocyte exposure to inhibitors of glucose metabolism, such as 2-deoxyglucose and iodoacetate [30] or knockout of Glut4 [5] not only decrease glycolysis but also result in smaller calcium transients, slowing of calcium decay and reduced contractility. Notably, these treatments produced prominent slowing of diastolic relaxation [31], which is characteristic of diabetic cardiomyopathy, a common clinical example of decreased cardiac glycolysis.

The ratios of reduced to oxidized forms of pyridine nucleotides NAD^+^ and NADP^+^ influence a plethora of functions in cells including cardiomyocytes [32]. The overall ratio of reduced to oxidized forms of pyridine nucleotides (NADPH plus NADH/NAD^+^ plus NADP^+^) was significantly elevated in both groups of TAC treated mice. Aon et al [33] proposed that cellular reductive status above or below an optimal range contributes to oxidative stress which damages cellular function. This may contribute to the dysfunction evident in all TAC treated mice. Overall reductive status was most severe in Mb-TAC hearts. This was due to a large increase in the NADPH/NADP^+^ ratio of Mb hearts, which developed only after TAC treatment. One major source of NADPH is the pentose phosphate pathway (PPP) which is controlled by the activity of glucose-6-phophate dehydrogenase (G6PDH) and availability of its substrate glucose-6-phosphate (G6P). We previously reported that G6P is elevated in Mb hearts [12] due to the downstream bottleneck in glycolysis produced by inhibition of PFK1. Despite increased G6P, there was no elevation in the NADPH/NADP^+^ ratio of Mb-sham mice. This may be due to the very low activity of G6PDH in normal hearts which minimizes PPP activity [34]. Heart failure elevates G6PDH activity [35] which in combination with the elevated G6P of Mb mice may account for the sharp rise in the NADPH/NADP^+^ ratio in Mb-TAC mice. NADPH is the substrate for superoxide producing NOX enzymes and its increase could contribute to the higher levels of 4HNE in Mb-TAC hearts. Elevated reductive status of pyridine nucleotides inhibits many steps in cardiac fuel metabolism [32] including glycolytic enzymes, pyruvate dehydrogenase, the TCA cycle and electron transport. This could contribute to reduced energy reserves of TAC hearts, especially Mb-TAC hearts. A limitation of these proposals is that we did not distinguish mitochondrial from cytoplasmic changes in pyridines which determines the specific enzymatic changes that can be altered by the greater reductive status of Mb-TAC hearts.

After TAC surgery, several molecular differences between Mb and FVB hearts developed which may contribute to the greater phenotypic response of Mb hearts to TAC. These differences included an increase in reductive capacity and increased oxidative stress. As noted above higher NADPH/NADP+ potentially stimulates superoxide production. Cardiac hypertrophy is associated with reduced rates of fatty acid oxidation due to the Randle cycle. In Mb hearts, lower glycolysis leaves the myocyte more dependent on fatty acid oxidation and this is associated with an increase in oxidative stress [36]. Higher levels of 4HNE protein adducts indicated more oxidative stress in Mb-TAC mice. Increased oxidative stress may not only stimulate cardiac hypertrophy but also promote fibrosis [37], [38], [39]. Furthermore, hypertrophy due to impaired glycolysis in the Glut4 knockout model was ameliorated by the antioxidant tempol [40], suggesting that oxidative stress may play a role in the hypertrophic response in most hearts with impaired glycolysis.

Several conclusions can be drawn from this study of mice with reduced cardiac F-2,6-P_2_. TAC surgery increased glycolysis in FVB hearts but not in Mb hearts that have much lower levels of F-2,6-P_2_. The inability to elevate glycolysis was associated with greater cardiac structural and functional changes, abnormal energetic status, higher reductive capacity and greater oxidative stress. Sham treated Mb hearts displayed no abnormalities. These results indicate that increasing cardiac glycolysis is an important adaptive response to pressure overload that requires a sufficient elevation in F-2,6-P_2_.

## References

[1] Bishop SP, Altschuld RA (1970) Increased glycolytic metabolism in cardiac hypertrophy and congestive failure. AmJPhysiol 218: 153–159. 10.1152/ajplegacy.1970.218.1.153 4243400

[2] Schwartz K, Boheler KR, de la BD, Lompre AM, Mercadier JJ (1992) Switches in cardiac muscle gene expression as a result of pressure and volume overload. AmJ Physiol 262: R364–R369. 1532697 10.1152/ajpregu.1992.262.3.R364

[3] Hamby RI, Zoneraich S, Sherman L (1974) Diabetic cardiomyopathy. JAMA 229: 1749–1754. 4278055

[4] Liao R, Jain M, Cui L, D’Agostino J, Aiello F, et al (2002) Cardiac-specific overexpression of GLUT1 prevents the development of heart failure attributable to pressure overload in mice. Circulation 106: 2125–2131. 12379584 10.1161/01.cir.0000034049.61181.f3

[5] Domenighetti AA, Danes VR, Curl CL, Favaloro JM, Proietto J, et al (2010) Targeted GLUT-4 deficiency in the heart induces cardiomyocyte hypertrophy and impaired contractility linked with Ca(2+) and proton flux dysregulation. J Mol Cell Cardiol 48: 663–672. 19962383 10.1016/j.yjmcc.2009.11.017

[6] Du XL, Edelstein D, Rossetti L, Fantus IG, Goldberg H, et al (2000) Hyperglycemia-induced mitochondrial superoxide overproduction activates the hexosamine pathway and induces plasminogen activator inhibitor-1 expression by increasing Sp1 glycosylation. ProcNatlAcadSciUSA 97: 12222–12226. 10.1073/pnas.97.22.12222 PMC17322 11050244

[7] Taylor M, Wallhaus TR, Degrado TR, Russell DC, Stanko P, et al (2001) An evaluation of myocardial fatty acid and glucose uptake using PET with [18F]fluoro-6-thia-heptadecanoic acid and [18F]FDG in Patients with Congestive Heart Failure. Journal of nuclear medicine: official publication, Society of Nuclear Medicine 42: 55–62. 11197981

[8] Kashiwaya Y, Sato K, Tsuchiya N, Thomas S, Fell DA, et al (1994) Control of glucose utilization in working perfused rat heart. Journal of Biological Chemistry 269: 25502–25514. 7929251

[9] Newsholme EA, Randle PJ (1964) Regulation of glucose uptake by muscle. 7. Effects of fatty acids, ketone bodies and pyruvate, and of alloxan-diabetes, starvation, hypophysectomy and adrenalectomy, on the concentrations of hexose phosphates, nucleotides and inorganic phosphate in perfused rat heart. BiochemJ 93: 641–651. 4220951 10.1042/bj0930641 PMC1214023

[10] Hue L, Beauloye C, Marsin AS, Bertrand L, Horman S, et al (2002) Insulin and ischemia stimulate glycolysis by acting on the same targets through different and opposing signaling pathways. JMolCell Cardiol 34: 1091–1097. 10.1006/jmcc.2002.2063 12392881

[11] Nascimben L, Ingwall JS, Lorell BH, Pinz I, Schultz V, et al (2004) Mechanisms for increased glycolysis in the hypertrophied rat heart. Hypertension 44: 662–667. 15466668 10.1161/01.HYP.0000144292.69599.0c

[12] Donthi RV, Ye G, Wu C, McClain DA, Lange AJ, et al (2004) Cardiac expression of kinase-deficient 6-phosphofructo-2-kinase/fructose-2,6-bisphosphatase inhibits glycolysis, promotes hypertrophy, impairs myocyte function, and reduces insulin sensitivity. Journal of Biological Chemistry 279: 48085–48090. 15331593 10.1074/jbc.M405510200

[13] Wu CD, Okar DA, Peng L, Lange AJ (2002) Decreasing fructose-2,6-bisphosphate leads to diabetic phenotype in normal mice. Diabetes 51: A319–A319.

[14] Facundo HT, Brainard RE, Watson LJ, Ngoh GA, Hamid T, et al (2012) O-GlcNAc signaling is essential for NFAT-mediated transcriptional reprogramming during cardiomyocyte hypertrophy. American journal of physiology Heart and circulatory physiology 302: H2122–2130. 22408028 10.1152/ajpheart.00775.2011 PMC3362113

[15] Liang Q, Carlson EC, Donthi RV, Kralik PM, Shen X, et al (2002) Overexpression of metallothionein reduces diabetic cardiomyopathy. Diabetes 51: 174–181. 11756338 10.2337/diabetes.51.1.174

[16] Liang Q, Donthi RV, Kralik PM, Epstein PN (2002) Elevated hexokinase increases cardiac glycolysis in transgenic mice. CardiovascRes 53: 423–430. 10.1016/s0008-6363(01)00495-3 11827693

[17] Watson LJ, Facundo HT, Ngoh GA, Ameen M, Brainard RE, et al (2010) O-linked beta-N-acetylglucosamine transferase is indispensable in the failing heart. Proc Natl Acad Sci U S A 107: 17797–17802. 20876116 10.1073/pnas.1001907107 PMC2955091

[18] Vandesompele J, De Preter K, Pattyn F, Poppe B, Van Roy N, et al (2002) Accurate normalization of real-time quantitative RT-PCR data by geometric averaging of multiple internal control genes. Genome biology 3: RESEARCH0034. 12184808 10.1186/gb-2002-3-7-research0034 PMC126239

[19] Wang J, Song Y, Elsherif L, Song Z, Zhou G, et al (2006) Cardiac metallothionein induction plays the major role in the prevention of diabetic cardiomyopathy by zinc supplementation. Circulation 113: 544–554. 16432057 10.1161/CIRCULATIONAHA.105.537894

[20] Lowry OH, Passonneau JV (1993) A flexible system of enzymatic analysis. New York: Academic.

[21] Wang Q, Donthi RV, Wang J, Lange AJ, Watson LJ, et al (2008) Cardiac phosphatase-deficient 6-phosphofructo-2-kinase/fructose-2,6-bisphosphatase increases glycolysis, hypertrophy, and myocyte resistance to hypoxia. Am J Physiol Heart Circ Physiol 294: H2889–2897. 18456722 10.1152/ajpheart.91501.2007 PMC4239994

[22] Donthi RV, Ye G, Wu C, McClain DA, Lange AJ, et al (2004) Cardiac expression of kinase-deficient 6-phosphofructo-2-kinase/fructose-2,6-bisphosphatase inhibits glycolysis, promotes hypertrophy, impairs myocyte function, and reduces insulin sensitivity. J Biol Chem 279: 48085–48090. 15331593 10.1074/jbc.M405510200

[23] Bernofsky C, Swan M (1973) An improved cycling assay for nicotinamide adenine dinucleotide. Anal Biochem 53: 452–458. 4351948 10.1016/0003-2697(73)90094-8

[24] Zerez CR, Lee SJ, Tanaka KR (1987) Spectrophotometric determination of oxidized and reduced pyridine nucleotides in erythrocytes using a single extraction procedure. Anal Biochem 164: 367–373. 3674385 10.1016/0003-2697(87)90506-9

[25] Neubauer S, Krahe T, Schindler R, Horn M, Hillenbrand H, et al (1992) 31P magnetic resonance spectroscopy in dilated cardiomyopathy and coronary artery disease. Altered cardiac high-energy phosphate metabolism in heart failure. Circulation 86: 1810–1818. 1451253 10.1161/01.cir.86.6.1810

[26] Neubauer S, Horn M, Cramer M, Harre K, Newell JB, et al (1997) Myocardial phosphocreatine-to-ATP ratio is a predictor of mortality in patients with dilated cardiomyopathy. Circulation 96: 2190–2196. 9337189 10.1161/01.cir.96.7.2190

[27] Marsin AS, Bertrand L, Rider MH, Deprez J, Beauloye C, et al (2000) Phosphorylation and activation of heart PFK-2 by AMPK has a role in the stimulation of glycolysis during ischaemia. CurrBiol %19 10: 1247–1255. 10.1016/s0960-9822(00)00742-9 11069105

[28] Kim M, Shen M, Ngoy S, Karamanlidis G, Liao R, et al (2012) AMPK isoform expression in the normal and failing hearts. Journal of molecular and cellular cardiology 52: 1066–1073. 22314372 10.1016/j.yjmcc.2012.01.016 PMC3327798

[29] Zhang P, Hu X, Xu X, Fassett J, Zhu G, et al (2008) AMP activated protein kinase-alpha2 deficiency exacerbates pressure-overload-induced left ventricular hypertrophy and dysfunction in mice. Hypertension 52: 918–924. 18838626 10.1161/HYPERTENSIONAHA.108.114702 PMC2760829

[30] Kockskamper J, Zima AV, Blatter LA (2005) Modulation of sarcoplasmic reticulum Ca2+ release by glycolysis in cat atrial myocytes. J Physiol 564: 697–714. 15695247 10.1113/jphysiol.2004.078782 PMC1464475

[31] Kagaya Y, Weinberg EO, Ito N, Mochizuki T, Barry WH, et al (1995) Glycolytic inhibition: effects on diastolic relaxation and intracellular calcium handling in hypertrophied rat ventricular myocytes. JClinInvest 95: 2766–2776. 10.1172/JCI117980 PMC295961 7769117

[32] Ussher JR, Jaswal JS, Lopaschuk GD (2012) Pyridine nucleotide regulation of cardiac intermediary metabolism. Circulation Research 111: 628–641. 22904042 10.1161/CIRCRESAHA.111.246371

[33] Aon MA, Cortassa S, O’Rourke B (2010) Redox-optimized ROS balance: a unifying hypothesis. Biochim Biophys Acta 1797: 865–877. 20175987 10.1016/j.bbabio.2010.02.016 PMC2891851

[34] Andres A, Satrustegui J, Machado A (1980) Development of NADPH-producing pathways in rat heart. The Biochemical journal 186: 799–803. 7396838 10.1042/bj1860799 PMC1161716

[35] Gupte SA, Levine RJ, Gupte RS, Young ME, Lionetti V, et al (2006) Glucose-6-phosphate dehydrogenase-derived NADPH fuels superoxide production in the failing heart. Journal of molecular and cellular cardiology 41: 340–349. 16828794 10.1016/j.yjmcc.2006.05.003

[36] Yamagishi SI, Edelstein D, Du XL, Kaneda Y, Guzman M, et al (2001) Leptin induces mitochondrial superoxide production and monocyte chemoattractant protein-1 expression in aortic endothelial cells by increasing fatty acid oxidation via protein kinase A. The Journal of biological chemistry. 276: 25096–25100. 10.1074/jbc.M007383200 11342529

[37] Lu Z, Xu X, Hu X, Lee S, Traverse JH, et al (2010) Oxidative stress regulates left ventricular PDE5 expression in the failing heart. Circulation 121: 1474–1483. 20308615 10.1161/CIRCULATIONAHA.109.906818 PMC3110701

[38] Takimoto E, Kass DA (2007) Role of oxidative stress in cardiac hypertrophy and remodeling. Hypertension 49: 241–248. 17190878 10.1161/01.HYP.0000254415.31362.a7

[39] Tanaka K, Honda M, Takabatake T (2001) Redox regulation of MAPK pathways and cardiac hypertrophy in adult rat cardiac myocyte. J Am Coll Cardiol 37: 676–685. 11216996 10.1016/s0735-1097(00)01123-2

[40] Ritchie RH, Quinn JM, Cao AH, Drummond GR, Kaye DM, et al (2007) The antioxidant tempol inhibits cardiac hypertrophy in the insulin-resistant GLUT4-deficient mouse in vivo. J Mol Cell Cardiol 42: 1119–1128. 17490678 10.1016/j.yjmcc.2007.03.900

